# Occurrence of Fatal and Nonfatal Adverse Outcomes after Heart Transplantation in Patients with Pretransplant Noncytotoxic HLA Antibodies

**DOI:** 10.1155/2013/519680

**Published:** 2013-07-29

**Authors:** Luciano Potena, Andrea Bontadini, Sandra Iannelli, Fiorenza Fruet, Ornella Leone, Francesco Barberini, Laura Borgese, Valentina Manfredini, Marco Masetti, Gaia Magnani, Francesco Fallani, Francesco Grigioni, Angelo Branzi

**Affiliations:** ^1^Cardiovascular Department, Academic Hospital S. Orsola-Malpighi, University of Bologna, Building 21, Via Massarenti 9, 40138 Bologna, Italy; ^2^Immunogenetics Unit, Transfusion Service, Academic Hospital S. Orsola-Malpighi, University of Bologna, Italy; ^3^Pathology Department, Academic Hospital S. Orsola-Malpighi, University of Bologna, Italy

## Abstract

HLA antibodies (HLA ab) in transplant candidates have been associated with poor outcome. However, clinical relevance of noncytotoxic antibodies after heart transplant (HT) is controversial. By using a Luminex-based HLA screening, we retested pretransplant sera from HT recipients testing negative for cytotoxic HLA ab and for prospective crossmatch. Out of the 173 consecutive patients assayed (52 ± 13*y*; 16% females; 47% ischemic etiology), 32 (18%) showed pretransplant HLA ab, and 12 (7%) tested positive against both class I and class II HLA. Recipients with any HLA ab had poorer survival than those without (65 ± 9 versus 82 ± 3%; *P* = 0.02), accounting for a doubled independent mortality risk (*P* = 0.04). In addition, HLA-ab detection was associated with increased prevalence of early graft failure (35 versus 15%; *P* = 0.05) and late cellular rejection (29 versus 11%; *P* = 0.03). Of the subgroup of 37 patients suspected for antibody mediated rejection (AMR), the 9 with pretransplant HLA ab were more likely to display pathological AMR grade 2 (*P* = 0.04). By an inexpensive, luminex-based, HLA-screening assay, we were able to detect non-cytotoxic HLA ab predicting fatal and nonfatal adverse outcomes after heart transplant. Allocation strategies and desensitization protocols need to be developed and prospectively tested in these patients.

## 1. Introduction

Short- and long-term mortality after heart transplantation (HT) has steadily decreased over time, reflecting the improvements in perioperative management and immunosuppressive therapy [1]. Nevertheless, even in the context of modern immunosuppressive strategies, the presence of preformed anti-human leukocyte antigens (HLA) antibodies (ab) in transplant candidates is still an important risk factor for allograft rejection and graft loss [2, 3].

In the last decade, the classical detection method based on complement dependent cytotoxicity (CDC) assays over a panel of leukocytes (panel reactive antibodies—PRA) has been replaced by more sensitive assays based on solid phase recognition of circulating ab [4]. These techniques allow detecting circulating anti-HLA ab in a higher number of patients than CDC assays, thus raising concerns regarding organ allocation and patient management during waiting list [5]. However, in what way the information from solid phase assays is translated into clinical practice to balance the risk of inappropriately delaying transplant on the one hand, or allocating organs likely to be affected by acute or chronic antibody mediated rejection on the other, is still a matter of investigation [4, 6]. Moreover, cost-effectiveness of the most sensitive techniques based on single antigen detection, which are also the most expensive, is poorly investigated in the heart transplant setting [7].

Seeking to provide a background for clinical decision making in patients with negative CDC, but with circulating HLA ab, we undertook this study to investigate the risk of fatal and nonfatal posttransplant adverse outcomes in a series of consecutive HT recipients who received the graft before the solid-phase technology become available in our center.

## 2. Methods

### 2.1. Study Design and Endpoints

We retested all the available pretransplant sera of the recipients who had proved negative in the classical PRA test and in CDC crossmatch with donor lymphocytes at the time of transplantation, between 2000, when we started routinely induction with thymoglobulins, and 2005, before solid phase assays were available in our laboratory. Thus, organ allocation or posttransplant management was not influenced by knowledge of circulating anti-HLA ab in these patients. 

**Figure 1:**
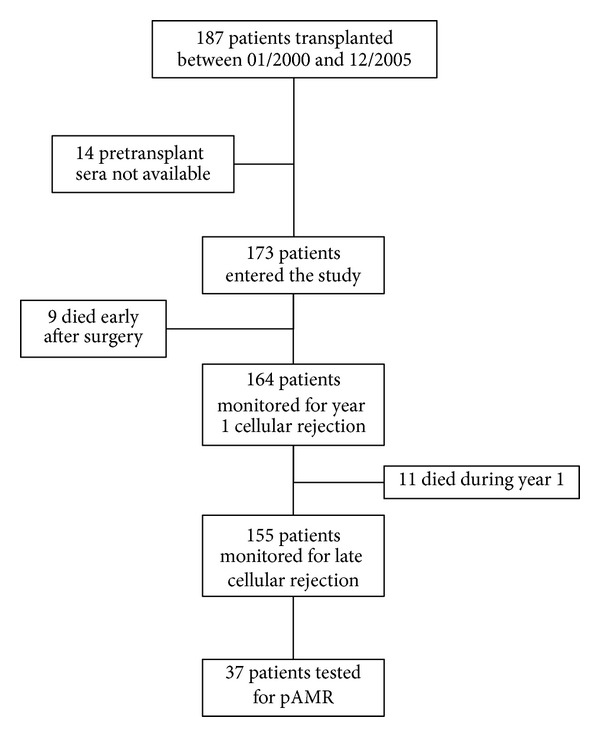
Study flow chart.

Clinical charts were reviewed to assess demography and study outcomes that comprised overall survival, early graft failure, early and late cellular rejection, and pathological antibody-mediated rejection (AMR) [8].  Figure 1 shows a flow chart of the study and the number of patients in whom it was possible to assess each of the outcomes. 

*Early graft failure* (EGF) was defined as a need for post-operative mechanical support (either extracorporeal membrane oxygenation (ECMO) or intra-aortic balloon pump (IABP)) or 30-day death/retransplant. 

#### 2.1.1. Cellular Rejection

All patients were routinely monitored for cellular rejection with scheduled endomyocardial biopsies (EMB) performed during the first five posttransplant years. EMB performed before 2006 were graded according to the 1990 ISHLT grading [9], while a new grading system [10] was followed for EMB performed from 2006 on. For study purposes, we report the incidence of clinically meaningful rejection as the occurrence of EMB graded 3A/2R or greater. We defined as *late rejection* the detection of a 3A/2R or greater grade after the first posttransplant year. 

#### 2.1.2. Antibody-Mediated Rejection

AMR was not routinely monitored in study patients. Following the first official consensus document dealing with the diagnosis of AMR in heart transplant recipients published in 2005 [10], only EMBs performed after 2005 were routinely screened for pathological AMR. Immunohistochemistry for C4d on paraffin embedded sections was performed only in biopsies with late cellular rejection, or histological picture suggestive of AMR, or in presence of hemodynamic compromise (i.e., capillary wedge pressure ≥18 mmHg, or drop in ejection fraction ≥15%, or symptoms of hear failure) [11]. Thus, we are able to report the incidence of pathological AMR (pAMR) only in the subgroup of patients who underwent complete immunohistochemistry stainings (Figure 1). Following the most recent ISHLT consensus criteria for the diagnosis of pAMR [8], EMB with full histological and immunohistochemistry AMR assessment have been reviewed and graded accordingly, by an experienced pathologist (O. Leone) who was blinded to the anti-HLA assessment. 

This study complies with national ethical rules and regulation for investigations on human subjects.

### 2.2. Antibody Detection

At time of listing, all patients were prospectively typed at the HLA-A, HLA-B, and HLA-DR loci by serological or molecular biology techniques and received antibody screening by classical PRA cytotoxicity assay, which resulted negative in all patients included in the current study [12]. At time of transplant, CDC donor crossmatch tested negative in all cases.

In all consecutive patients with serum availability (Figure 1), we re-tested the sera to identify IgG anti-HLA reactivity by a bead-based screening assay on the Luminex platform (LABScan 100). We used the Labscreen mixed kit (One Lambda, Inc., Canoga Park, CA, USA) following the manufacturer's instructions. Briefly, Labscreen simultaneously detects class I and class II antibodies by a pool of microbeads coated with purified class I an class II antigens. Collectively, there are beads representing 96 class I (A, B, C) and 56 class II (DR, DQ, DP) antigens/alleles. Patient serum was incubated with the beads at room temperature in the dark for 30 minutes, washed twice and subsequently incubated with phycoerythrin-conjugated anti-human IgG in the dark with gentle shaking for 30 minutes, washed twice, and analyzed in the Luminex platform. In the assay, antibodies are detected by the fluorescence intensity (FI) of each HLA antigen coated bead. Normalized mean fluorescent indexes (MFI) from Fusion software (One Lambda, Inc.) were used to assign positive antibodies (MFI > 500). This assay detects all HLA IgG-binding antibodies and is unable to discriminate single antigen type [13].

While not providing HLA specificity of the detected antibody, this test is available at a cost of 30 €/test, as opposed to the single bead HLA assay, which allows detecting HLA specificity for about 600 €/test.

### 2.3. Immunosuppressive Therapy

Standard immunosuppressive protocol consisted in induction with low-dose thymoglobulins (0.75 to 1 mg/Kg/day for 3–5 days) followed by maintenance therapy based on cyclosporine, azathioprine (AZA), and prednisone (0.2 mg/Kg/d, tapered off in patients with no rejection). After November 2004, AZA was substituted by mycophenolate mofetil (MMF) for routine practice. 

Cellular rejection graded 3A (2R) or greater was treated with 1 g/day for three days with methyl-prednisolone i.v., or with thymoglobulin if associated with hemodynamic compromise or steroid resistant. 

Treatment of AMR was not standardized and often depended on treating physician. Mostly, patients with pathological signs but no symptoms were not treated or received uptitration of maintenance immunosuppression. Patients with pAMR and clinical or instrumental signs of graft dysfunction received plasmapheresis and IVIG. One case of relapsing AMR received rituximab. This strategy was in line with current practice in most of transplant centers, as reported by a recent survey conducted within ISHLT [14].

### 2.4. Statistical Analysis

Continuous variables are reported as means ± standard deviation, categorical variables as numbers (percentages). Student's *t*-test or Chi-Square tests were used as appropriate to assess differences between groups. Overall survival was assessed by Kaplan-Meier method and differences between groups with log-rank test. The association of anti-HLA ab with study outcomes was adjusted for potential confounders by using Cox proportional hazards method (for all-cause death and cellular rejection) or by logistic regression (for EGF). A *P* value < 0.05 was considered as significant. 

## 3. Results

### 3.1. Study Population and Prevalence of Anti-HLA ab

Out of the 173 recipients fulfilling inclusion criteria, 32 (18%) were found to have circulating pretransplant HLA ab. Of these, 28 (87%) showed ab against class I HLA antigens, 16 (50%) against class II, and 12 (38%) tested positive for ab against both class I and class II HLA antigens. As expected, female patients presented HLA ab more often than males (Table 1), with 10 (83%) of them reporting previous pregnancies. While not significantly associated with HLA ab in the overall study population (Table 1), previous heart surgery appeared to favor HLA sensitization in males, because HLA ab was detected in 10 (21%) of the 47 patients with previous heart surgery versus in 9 (9%) of the 96 without (*P* = 0.05). MMF, routinely introduced after November 2004, was less often used in patients with HLA ab. During the study period, none of the recipients had received a ventricular assist device implant before transplant.

### 3.2. Anti-HLA ab and All-Cause Mortality

**Figure 2:**
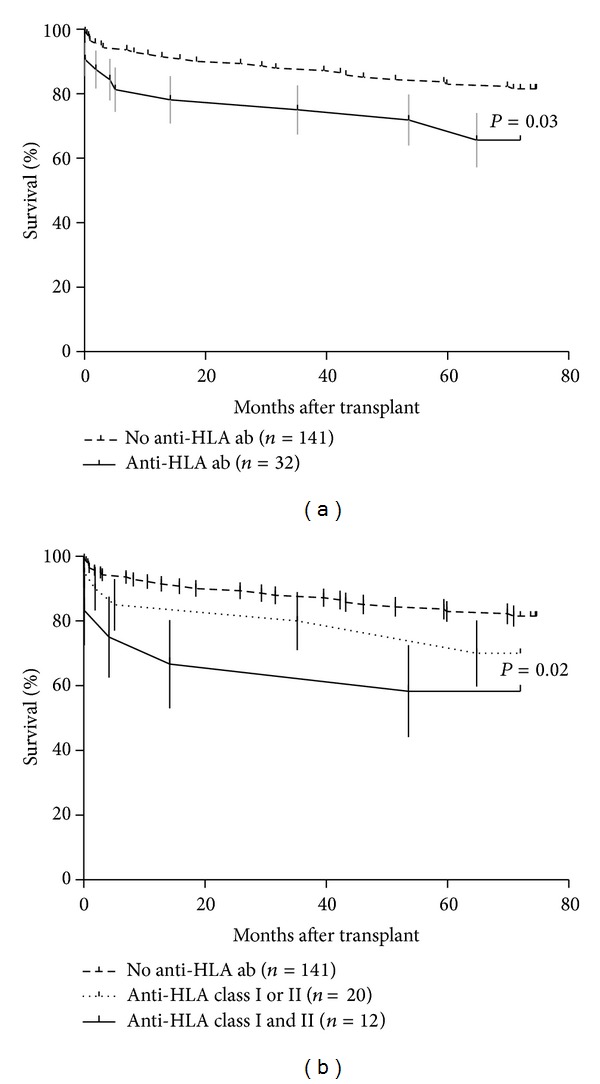
Kaplan-Meier estimate survival in patients with HLA ab. (a) Overall survival in patients with (solid line) and without (dotted line) pretransplant HLA ab. Vertical lines indicate standard errors of the survival estimate. (b) Overall survival according with HLA antibodies against either class I or II alone (dotted line), both class I and II (solid line), or no HLA antibodies. Vertical lines indicate standard errors of the survival estimate.

Overall, during the 6-year study follow-up, 36 patients died and one was retransplanted, accounting for an estimated graft survival of 78 ± 3%. Patients with pretransplant HLA ab had significantly worse survival than those without (65 ± 9 versus 82 ± 3%; *P* = 0.02; Figure 2(a)). Furthermore, patients with both class I and class II HLA showed even poorer estimated survival (55 ± 15%; Bonferroni *P* = 0.04 versus ab negative patients) while those with either anti class I or class II alone, showed an intermediate estimated survival (Figure 2(b)). In the majority of cases, death was attributed to graft-related causes (18 (49%) including CAV, sudden death, rejection, and unspecified graft failure), followed by infection (21%), malignancy (11%), and others (16%). Causes of death did not appear different between patients with and without anti-HLA ab (*P* = 0.9).

Multivariable analysis showed that the negative effect of pretransplant HLA ab on survival persisted after adjusting for recipient's gender and for pretransplant cardiac surgery, the two factors we found to be associated with anti HLA ab development (adjusted RR (95% CI) = 2.4 (1.03–5.12); *P* = 0.04). Of note, the use of MMF as well did not appear to influence the negative effect of anti-HLA ab on survival (adjusted RR (95% C.I.) = 2.23 (1.04–4.52); *P* = 0.04). 

### 3.3. Anti-HLA and Other Graft-Related Outcomes

**Figure 3:**
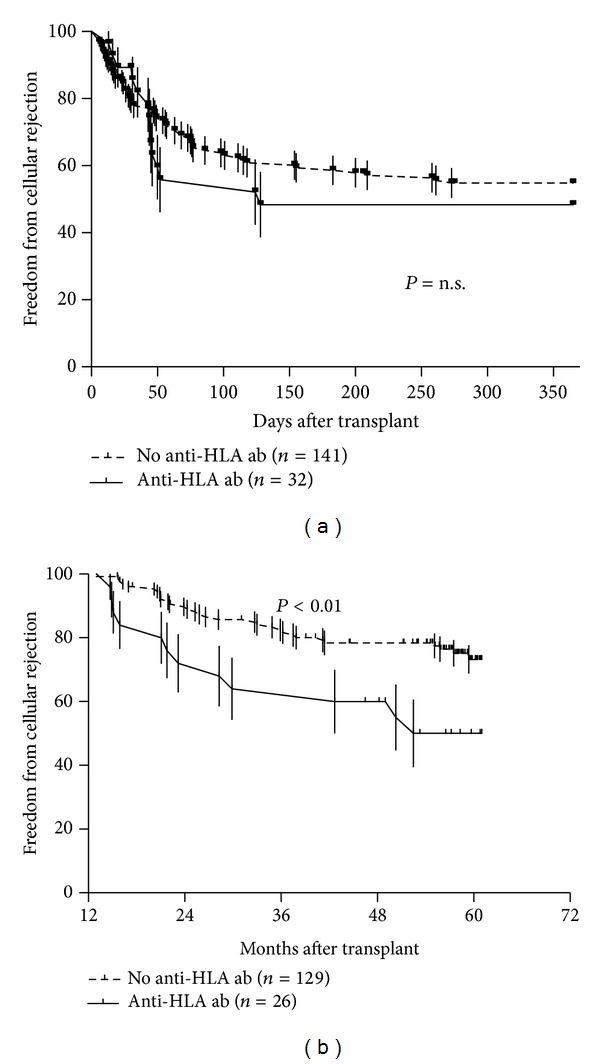
Kaplan-Meier estimate of freedom from cellular rejection. (a) Survival free from cellular rejection graded ≥3A/2R on surveillance endomyocardial biopsies performed during year 1 in patients with or without pretransplant HLA ab. (b) Survival free from cellular rejection graded ≥3A/2R on surveillance endomyocardial biopsies performed after year 1 in patients with or without pretransplant HLA ab.

EGF occurred in 18 (10%) patients (10 in-hospital deaths, 5 ECMO, 3 IABP). Patients with any positive anti-HLA ab (22% versus 8%; *P* = 0.03, Figure 3), as well as female recipients (31% versus 7%; *P* < 0.01), were more likely to develop EGF. After factoring both variables into a multivariable logistic model, only female gender resulted independently associated with an increased risk for EGF (adjusted OR (95% CI) = 4.84 (1.54–14.9); *P* < 0.01). 

Cellular rejection was routinely monitored with scheduled endomyocardial biopsies for the first 5 years after transplant. As shown in Figure 1, 164 patients survived long enough to receive a biopsy during year 1, and 155 patients received at least one biopsy during years 2 to 5. While pretransplant anti-HLA ab did not seem to influence year 1 CR, they were associated with a higher incidence of late CR (Figure 3). Of note, multivariable analysis showed that recipient's female gender or therapy with MMF did not appear to influence the impact of pretransplant anti-HLA ab on the risk of late CR (adjusted RR (95% C.I.) = 2.49 (1.18–4.97); *P* = 0.02). 

**Figure 4:**
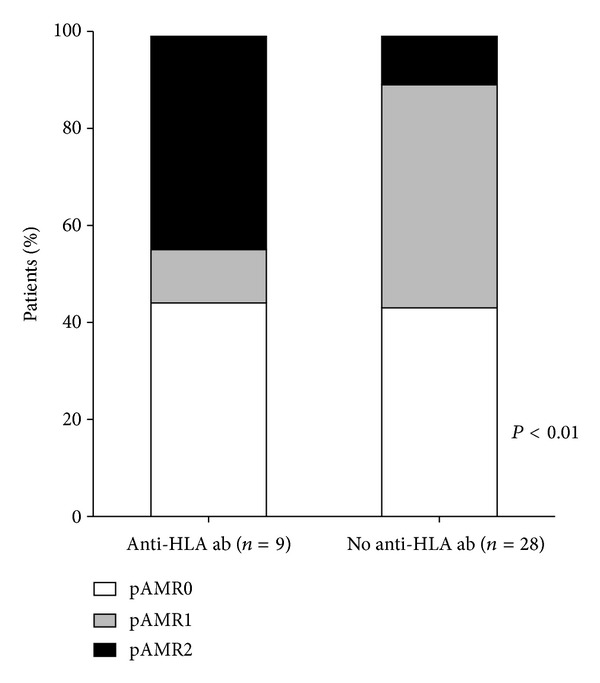
Anti-HLA ab and pAMR. Distribution of pAMR grades in patients with and without pretransplant HLA ab.

Complete histological and immunohistochemistry diagnosis of pAMR was available in only 37 patients (Figure 1) in EMB performed 62 ± 27 months after transplant. Of those, 9 (24%) had pretransplant anti-HLA ab.  Figure 4 shows the distribution of pAMR grade between the two groups of patients. Interestingly, while the prevalence of a negative biopsy was similar between the study groups, pAMR grades 1 and 2 differed significantly, with pAMR 2 being more frequent in patients with anti-HLAab (Bonferroni adjusted *P* = 0.04).

## 4. Discussion

Since the late sixties, pretransplant anti-HLA sensitization has been associated with high risk of graft loss, so it was deemed unacceptable to allocate organs across a positive crossmatch [15]. Traditional PRA assays allow us to estimate the probability of listed patients receiving a graft expressing HLA antigens which the patients are primed against, limiting organ allocation only after a negative CDC prospective crossmatch (usually for patients with PRA >10%). Indeed, elevated PRA was found to be an independent risk factor for graft loss in a large number series of heart transplant recipients [3]. The use of the more sensitive solid phase assays, which allows detection of noncytotoxic circulating anti-HLA ab [4], and the increased referral of patients with previous cardiothoracic surgery (i.e., extreme heart failure surgery and mechanical circulatory supports) markedly enhanced the rate of heart transplant candidates detected with anti-HLA antibodies. To what extent these pre-formed antibodies identify unacceptable risk for organ allocation is still a matter of debate [2, 16–18], and current consensus in heart transplantation is mainly based on a few single center experiences [16]. Moreover, the high cost of the most sensitive techniques may limit the applicability of a thorough anti-HLA ab screening in a setting of limited healthcare resources. 

In this study, by re-testing with a low-cost Luminex-based assay the pretransplant sera from a series of consecutive recipients who had a negative PRA as evaluated with CDC testing, we identified a subgroup of patients with noncytotoxic anti-HLA ab who showed increased risk of death and nonfatal adverse events during the posttransplant follow-up, independently from immunosuppressive strategy and other possible confounders.

In line with other published series, we found that about 1 in every 5 patients showed either class I or II noncytotoxic anti-HLA ab before transplantation, [2] with female gender and previous cardiothoracic surgery being the major risk factors for their development. Detection of anti-HLA ab and in particular of antibodies against both class I and II HLA was independently associated with increased risk of all-cause mortality, up to 6 years after transplant. Lack of differences in specific causes of death between sensitized and not sensitized patients raises the hypothesis that pretransplant anti-HLA antibodies may represent a generic marker of posttransplant risk, not only involved in the specific immunologic pathways of antibody-mediated rejection. This concept is supported by the finding that despite eliciting more powerful immunosuppression, the use of MMF instead of azathioprine did not influence the mortality risk associated with anti-HLA ab. Nevertheless, we cannot exclude that limited sample size, and timing of the deaths observed in the anti-HLA ab patients, mostly occurring early after transplant, could explain lack of MMF benefit. 

Although early mortality appeared to be the major threat, late cellular rejection, as detected by protocol biopsies, occurred more frequently in sensitized patients surviving longer than one year after transplant. This finding supports the concept of a strict interplay between circulating antibodies and cellular rejection, with alloantibodies behaving as opsonins facilitating T-cell activation and limiting the development of tolerance towards the graft [19]. In addition, as a possible consequence for clinical practice, detection of pretransplant HLA ab may help to identify patients in whom biopsy surveillance may be required even after the first posttransplant year [20].

Only a small group of patients was assessed for pathological signs of AMR, in biopsies performed after 2005, [10] and the lack of comprehensive pathological and serological AMR monitoring in most of the study patients reduces the ability of our results to provide robust correlations between the pathological AMR grading and HLA ab. Nevertheless, in a subgroup of 37 patients we were able to find suggestive evidence correlating pAMR grading with pretransplant HLA ab. In particular (Figure 4), circulating HLA ab appeared to be associated with pAMR 2 (i.e., coexistence of histological damage and C4d activation) but with EMB with either histological or C4d criteria only (i.e., pAMR1). To our knowledge, this is the first observation linking the new pathological classification to serological markers of AMR [8].

### 4.1. Study Limitations

Retrospective design may represent a limitation of this study. However, by re-testing sera of patients who at the time of transplant were managed without the awareness of HLA antibodies, we provide an analysis of the effect of HLA pre-sensitization on outcome that is unbiased by medical treatment or organ allocation approach. Although allowing the simultaneous detection of noncytotoxic class I and class II HLA ab, the solid-phase assay used in this study does not provide single HLA antigen specificity, restraining us from providing an analysis of the impact of donor-specific antibodies, as opposed to any anti-HLA ab. We believe that this potential study limitation is counterbalanced by the low-cost of this assay that retains the ability to independently stratify posttransplant prognosis, helping to direct resources to selected group of high-risk patients.

## 5. Conclusions

This observational study shows that noncytotoxic HLA antibodies identify heart transplant candidates at high risk of death, early graft failure, and of late cellular and antibody-mediated rejection, underscoring the need for an accurate screening of HLA ab in heart transplant candidates. The ability of a low-cost assay to stratify prognosis may help to increase the feasibility of HLA ab detection even in a setting of low-resource healthcare systems. In addition, in a subgroup of patients, we provide first-time association between HLA ab and the recent pathological grading of AMR. Although prospective validation of specific strategies to manage sensitized heart transplant candidates is still lacking, our findings support the concept that approaches involving desensitization protocols, [21] allocation based on virtual crossmatch, [22], and customized immunosuppressive strategies may improve posttransplant outcome in these patients.

## Figures and Tables

**Table 1:** Baseline characteristics according with HLA antibodies.

	Anti-HLA antibodies (*n* = 32)	No anti-HLA antibodies (*n* = 141)	*P*
Age (y)	55 ± 13	52 ± 13	0.2
Sex (females %)	12 (38%)	15 (11%)	<0.01
Pre-transplant CAD (%)	16 (50%)	65 (46%)	0.7
Donor age (y)	35 ± 12	34 ± 13	0.7
Donor sex (females %)	5 (16%)	40 (29%)	0.1
Donor cause of death (stroke %)	15 (48%)	45 (33%)	0.1
Pre-transplant heart surgery (%)	13 (41%)	42 (30%)	0.2
Cold ischemic time	187 ± 41	190 ± 51	0.7
Started on MMF versus AZA (%)	3 (9%)	32 (24%)	0.05
